# Prediction was predictable from human brain activity in fronto-parietal cortex

**DOI:** 10.1186/1471-2202-14-S1-P181

**Published:** 2013-07-08

**Authors:** Yumi Shikauchi, Ishii Shin

**Affiliations:** 1 Graduate School of Informatics, Kyoto University, Gokasho, Uji, Kyoto 611-0011, Japan; 2 ATR Neural Information Analysis Laboratories, 2-2-2 Hikaridai Seika-cho, Sorakugun, Kyoto, 619-0288 Japan

## 

In partially observable environments, prediction of the future is a key to make appropriate decisions [1,2]. Whether or not to open the door (decision making) would depend on whether one could expect someone is behind the door (prediction) even in a well-known environment such as an office. Such interpolation of pre-observed information was obtained by integrating the past observations and the environment model. Recent evidence suggests that such model-based decision-making activates fronto-parietal cortex [3-6]. Despite popularity of neural decoding, research on mapping from neural signals into prediction of pre-observed information has rarely been reported.

Here, we show that prediction decoding is possible from individuals' functional magnetic resonance imaging (fMRI) activity. We asked four healthy subjects to perform a scene prediction task, which required discriminating a true next scene in a three-dimensional navigation environment. We decoded the predicted scene consisting of three views; forward-center (fC), forward-left (fL) and forward-right (fR), information from fMRI activity in anatomically defined region of interests (ROIs): lateral prefrontal cortex (LPF), medial prefrontal cortex (MPF) and parietal cortex (PC). The decoding analysis was conducted individually for each subject and ROIs and the performance values across subjects were then averaged.

All subjects carried out the scene prediction task 92.6% ± 3.2% correct and 4.8% ± 3.5% incorrect. Using a leave-one-trial-out procedure, we decoded individuals predicted scene per view from the fMRI data of correct trials. When using PC activity, these decoders allowed us to read out predicted all views (fC: 70.94%; p < 0.01 × 10^-1^, fL: 60.43%; p < 0.01, fR: 65.18%; p < 0.05). In contrast, above-chance performance was obtained fC view only in LPF and MPF (LPF: 67.78%; p < 0.01 × 10^-3^, MPF: 62.97%; p < 0.05). Remarkably, PC bit decoders that trained by correct trials can also read out incorrect scene selected by the subject (fC: 66.77%; p < 0.01, fL: 58.19%; p = 0.43, fR: 75.43%; p < 0.05 × 10^-1^).

In this study, we demonstrated decoding of scene prediction from individuals fMRI activity. Because fC is the most important view for the subjects to determine the next motion, the fC decoders show the high decoding accuracy for all ROIs. These results suggest that prediction could be performed in the fronto-parietal network such to reflect the degree of contribution to the subsequent decision-making. Our findings have outlined the decision making system employed in complicated environments, and implied useful characters of decoders which can be used for brain machine interface of practical navigation systems.
